# Acute Thrombotic Occlusion of Right Coronary and Left Circumflex Coronary Arteries in a Patient with Antiphospholipid Syndrome: Successful Stent Implantation

**DOI:** 10.1155/2010/198594

**Published:** 2010-10-26

**Authors:** Serdar Biceroglu, Muge Ildizli Demirbas, Mustafa Karaca, Murat Yalcin, Hasan Yilmaz

**Affiliations:** ^1^Cardiology Department, Atakalp Heart Hospital, 1418 sk, no.18 Kahramanlar, 35200 Izmir, Turkey; ^2^Cardiology Department, İzmir Military Hospital, 35600 Izmir, Turkey

## Abstract

Antiphospholipid syndrome is a rare disorder. Acute myocardial infarction is uncommon among these patients. Here we report a case of a 44-year-old man with antiphospholipid syndrome admitted for acute inferior myocardial infarction. Performed coronary angiography revealed that both the right coronary and the left circumflex coronary arteries were occluded by thrombi. We successfully performed primary angioplasty and stent implantation for both of the occluded coronary arteries. In the literature, this is the first case with antiphospholipid syndrome in which primary coronary angioplasty with stent implantation was successfully performed for two coronary arteries with acute thrombotic occlusion.

## 1. Background

Antiphospholipid syndrome is a thrombotic disorder characterised by antiphospholipid antibodies. Clinical features are thrombosis, thrombocytopenia, and recurrent fetal loss [1]. This syndrome has the clinical manifestations of systemic thrombotic disorders, including recurrent deep vein thromboses, pulmonary thromboembolisms, and brain strokes. Antiphospholipid syndrome has systemic vascular thrombotic manifestations and these thromboses often occur and recur in multiple organs. Coronary events have been described to occur in approximately 5% of patients with APS, especially patients under age 45 [2].

The diagnosis of antiphospholipid syndrome is defined by the Sapporo criteria which were recently revised. These criteria require a clinical manifestation of one or more episodes of thrombosis or thrombotic-related events and the laboratory confirmation of an antiphospholipid antibody on two or more occasions at least 12 weeks apart. Notably, thrombotic events related to pregnancy, such as unexplained fetal loss or premature births related to preeclampsia, eclampsia, or placental insufficiency, are important clinical criteria. There are two basic types of antiphospholipid antibodies: the anticardiolipins (ACL) and the anticoagulant antibodies (LAC). The rationale for the time limitations on the presence of antiphospholipid antibodies comes from the fact that there are such antibodies present, frequently transiently, in up to 4%-5% of otherwise healthy individuals. They can also be seen in the setting of many infectious diseases, such as mumps, rubella, and malaria. Approximately one third of patients with systemic lupus erythematosus (SLE) have an antiphospholipid antibody. Not all antiphospholipid antibodies in the general population cause thrombotic complications. Approximately 50%–70% of patients with SLE with antiphospholipid antibodies will develop antiphospholipid syndrome. The incidence of antiphospholipid syndrome in patients without SLE is not well established. The characteristics that are linked to higher thrombotic complications include higher antibody titers, presence of the LAC antibody, and a history of thrombosis [3–8].

Although patients with antiphospholipid syndrome often exhibit positive lupus anticoagulant activity, they uncommonly present the typical clinical findings of SLE which are diagnostic. Thus, antiphospholipid syndrome without the clinical features of SLE is called primary antiphospholipid syndrome [9].

Acute myocardial infarction is not common among patients with this syndrome. In the literature, there are cases that are successfully treated by thrombolytic therapy, balloon angioplasty, and stenting but there is not enough experience about thrombus aspiration [10]. Here we report a case of a 44-year-old man with antiphospholipid syndrome admitted for acute inferior myocardial infarction. He did not have major traditional risk factors except that he smoked five cigarettes a day.

## 2. Case Report

**Figure 1:**
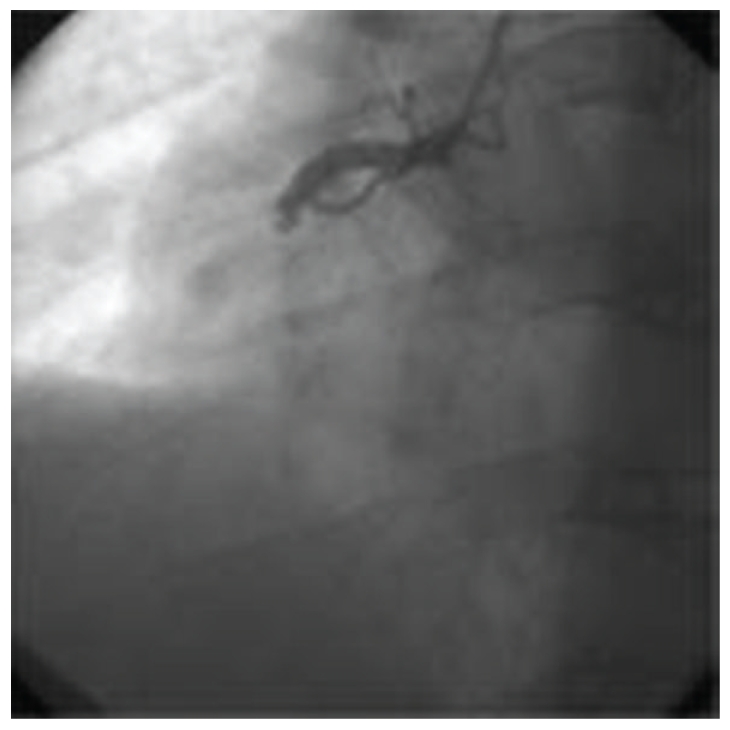
Occluded right coronary artery by trombi.

**Figure 2:**
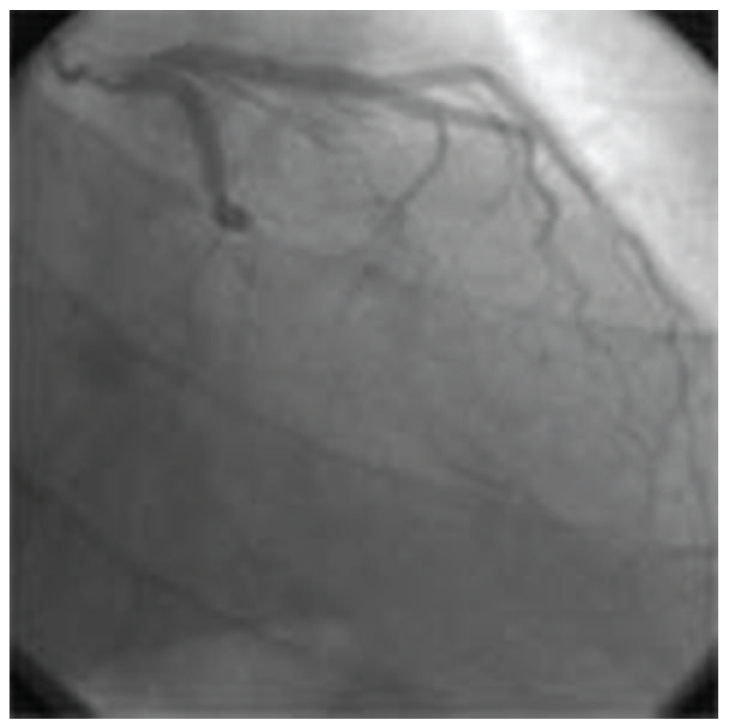
Occluded left circumflex coronary artery by trombi.

The patient admitted to the emergency service with typical chest pain persisting for 3 hours. Electrocardiography demonstrated >2 mm ST segment elevation in inferior leads. He was immediately transferred to the catheter laboratory for a primary angioplasty. Performed coronary angiography revealed that both the right coronary and the left circumflex coronary arteries were occluded by thrombi (Figures 1 and 2).

Antiphospholipid syndrome was remarkable in his medical history. He was diagnosed as antiphospholipid syndrome eight years ago after two attacks of deep vein thrombosis with positive anticardiolipin antibody. After this attack, he was followed at a rheumotology clinic and his anticardiolipin antibody was still positive at the third month, first and second years of the initial diagnosis. Proper anticoagulant treatment was given at that time. There was no positive family history of thoromboss and the patient did not have any other manifestations of antiphospholipid antibody syndrome after these two attacks. However, he stopped taking warfarin 6 weeks ago due to a dental procedure and delayed to initiate warfarin therapy after his dental treatment was completed. The INR level was less than 1.6 during this dental procedure.

**Figure 3:**
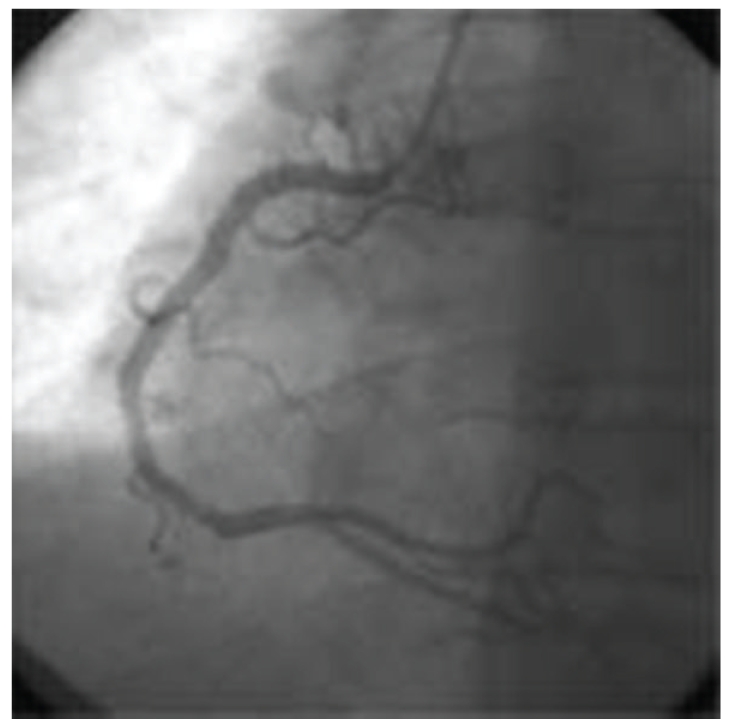
Right coronary artery angiogram after successful stent implantation.

**Figure 4:**
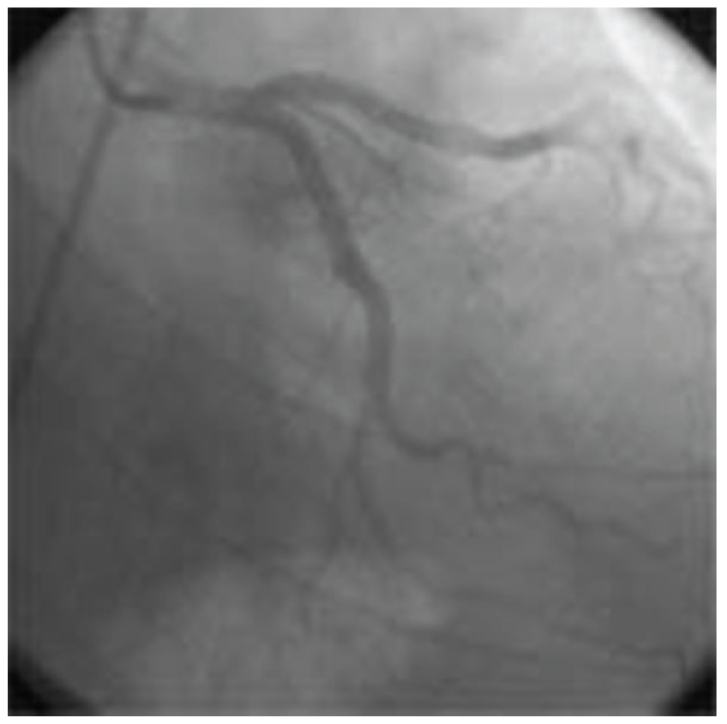
Left circumflex coronary artery angiogram after successful stent implantation.

Primary angioplasty and stent implantation for both the right coronary and the left circumflex coronary arteries were successfully performed. After stent implantation, coronary blood flow was TIMI III grade within both arteries (Figures 3 and 4).

In coronary care unit, intravenous heparin infusion was performed for the next 24 hours followed by warfarin (5 mg daily). The patient also received clopidogrel (75 mg daily following a 600 mg loading dose), aspirin (300 mg daily), metoprolol (50 mg daily), atorvastatin (20 mg daily), and ramipril (2.5 mg daily) after stent implantation.

The patient was consulted with rheumotology and hematology clinic in coronary care unit and the anticardiolipin antibody was positive.

Echocardiograpic examination did not reveal an intracardiac thrombus that could be a source for coronary embolism. Inferior and lateral wall segments of the left ventricle were moderately hypokinetic. Ejection fraction was 50% and there was mild mitral regurgitation.

After a four-day period, the patient was discharged from the hospital on clopidogrel (75 mg daily), warfarin (5 mg daily), metoprolol (50 mg daily), atorvastatin (20 mg daily), and ramipril (2.5 mg daily) therapy. INR level was 2.3 at the time of discharge. The patient was called for routine follow-up controls at the third and sixth months, he was followed up by rheumotology and hematology clinics. He was asymptomatic and had a negative exercise stress test at his sixth month of followup; therefore, coronary angiography was not performed. Also the anticardiolipin antibody was positive at sixth month.

## 3. Discussion

The most common clinical features of antiphospholipid syndrome are recurrent arterial or venous thrombotic events such as deep vein thrombosis, pulmonary thromboembolism, and stroke. However, coronary embolism due to this syndrome is not common [11]. Thus antiphospholipid syndrome may cause acute myocardial infarction due to thrombotic occlusion of coronary arteries. Since it is originated from a thrombotic occlusion, thrombolytic therapy was reported to be a rationale treatment option for such cases in the literature [12–14]. However, primary coronary angioplasty and stent implantation are currently an effective way of myocardial revascularization by opening an infarct related artery and it is the treatment of choice in definite clinical circumstances. In the literature, there were a few cases of antiphospholipid syndrome that were treated by coronary angioplasty in the setting of acute myocardial infarction until the year 1998 but the results were not successful [15]. The first successful primary angioplasty was reported by Takeuchi et al. in 1998 in a patient with antiphospholipid syndrome. Jankowski et al. reported the first successful stent implantation in patient with antiphospholipid syndrome in the same year [16]. In the year 2004, Prokop et al. reported the first patient with antiphospholipid syndrome presenting with acute myocardial infarction that was related to the thrombotic occlusion of two coronary arteries like in our case. In that case, both the left anterior descending and circumflex coronary arteries were occluded [17]. They became obligated to perform thrombolytic therapy following the failure of primary angioplasty.

Since stent implantation may probably trigger acute thrombosis in patient with antiphospholipid syndrome, only the balloon dilatation of the lesion may be an alternative choice. Also, thrombectomy aspiration device could have been an alternative catheter to stent implantation or an adjunctive catheter for baloon angioplasty without stenting. Tamhene et al. found that thrombectomy devices appear to improve markers of myocardial perfusion in patients undergoing primary percutaneous coronary interventions, with no difference in overall 30-day mortality but an increased likelihood of stroke [18]. Using thrombectomy aspiration device for better clinical outcome is only an assumption for this case. After baloon angioplasty, we had to implant stents in order to avoid restenosis and also because of the irregular vessel wall image in angiograms. Since the risk of thrombosis increases with fresh metal from a stent, antithrombotic therapy following stent implantation gains importance in such patients. In our case, we initiated acetylsalicylic acid, clopidogrel, and heparin infusion to prevent acute thrombosis. Use of glycoprotein IIb/IIIa inhibitors that are recommended following primary coronary angioplasty for ST elevated myocardial infarction might be used as an adjunctive treatment. However, we do not have enough experience in cases with antiphospholipid syndrome.

It seems that this is the first case in which primary coronary angioplasty with stent implantation was successfully performed to total occlusions in two coronary arteries induced by antiphospholipid syndrome. Although the balloon dilatation may seem rationale, we successfully performed stent implantation in this patient. As a conclusion it is very important to initiate and to maintain the antithrombotic drug therapy following coronary angioplasty and stent implantation in patients with antiphospholipid syndrome.
